# Predictors for adoption of e-learning among health professional students during the COVID-19 lockdown in a private university in Uganda

**DOI:** 10.1186/s12909-022-03735-7

**Published:** 2022-09-10

**Authors:** Alimah Komuhangi, Hilda Mpirirwe, Lubanga Robert, Florence Wamuyu Githinji, Rose Clarke Nanyonga

**Affiliations:** Clarke International University, Kampala, Uganda

**Keywords:** E-learning adoption, Health professional students, Coronavirus diseases 2019 lockdown

## Abstract

**Background:**

During the recent Coronavirus pandemic, many universities realized that the traditional delivery of educational content was not adequate in the context of imposed restrictions. Adoption of e-learning was one obvious way to foster continuity of learning. Despite its rapid implementation during the lockdown in Uganda, it was not known whether health professional students were willing to adopt e-learning as a way to foster continuity of learning. We, therefore, adopted a Technology Acceptance Model to determine the predictors for the adoption of e-learning using learner and information technology variables.

**Methods:**

A cross-sectional study among 109 health professional students ≥18 years of age at Clarke International University was conducted. Adoption of e-learning was measured as a self-report. Data were obtained using a smart survey and descriptively summarized. The differences in the study outcome were compared using the chi-square test. The factors that independently influenced the adoption of e-learning were determined using binary logistic regression and reported as adjusted odds ratios (aORs) with a 95% confidence interval (CI).

**Results:**

Of the 109 respondents, 71 (65.1%) adopted e-learning. Our data showed low odds of adoption of e-learning among participants in first year (aOR, 0.34: 95%CI, 0.14–0.79), low e-learning expectations (aOR, 0.01: 95%CI, 0.01–0.34), no confidence in using IT devices (aOR, 0.16: 95%CI, 0.00–0.77), no prior experience in e-learning (aOR, 0.11: 95%CI, 0.02–0.68), not considering e-learning flexible (aOR, 0.25:95%CI, 0.08–0.86) and high cost of internet (aOR, 0.13: 95%CI, 0.02–0.84).

**Conclusion:**

We identified predictors of e-learning adoption which include having completed at least 1 year of study, high e-learning expectations, confidence in using IT devices, prior experience in e-learning, considering e-learning to be flexible and internet access. This information can be used by universities to enhance infrastructure and prepare potential e-learners.

**Supplementary Information:**

The online version contains supplementary material available at 10.1186/s12909-022-03735-7.

## Background

E-learning (electronic learning) emerged as one of the ways to foster education continuity during the Coronavirus diseases 2019 pandemic. Worldwide, this new paradigm of the learning market has a growth rate of 35.6% [1]. E-learning modes include online learning, virtual learning, distance education, m-learning, and learning management systems [2]. In the developing world, ministries of education in different countries recommended or made it mandatory to implement online learning at all school levels [3]. E-learning initiatives are strongly tied to information and communication technologies (ICT) [4]. ICT provides an advanced e-learning environment to stimulate and enhance teaching and self-directed learning [5].

The Coronavirus diseases 2019 pandemic introduced the need for less human physical contact to prevent its spread in what is called “social distancing” [6]. This underscored the strong need for e-learning and for continued academic operations both of which could be achieved at a distance. In September 2020, during the protracted school lockdown, the Uganda National Council of Higher Education (NCHE) established the Emergency Open Distance e-Learning (ODeL) strategies for health professional students to continue learning remotely. The introduction of e-learning was not intended to replace the traditional face-to-face classroom teaching, but to provide remedial education strategies and new opportunities for continued learning during the Coronavirus diseases 2019 lockdown in Uganda [7].

Given the scarcity of health professional workers in the control of the ongoing Coronavirus diseases 2019 pandemic, e-learning needed to be adopted in universities to hasten the continuation of health professional training [7]. The rapid shift to e-learning introduced many health professional students to new ways of learning in virtual environments, as well as the use of e-learning tools and resources. Students in developing countries face significant economic limitations, weak internet connectivity, lack of knowledge of ICT usage all of which impact their readiness to adopt e-learning [1, 8] In Uganda, documented challenges include students’ lack of awareness and skills in using e-learning platforms, access to ICT tools, internet costs and connectivity [2, 3, 9].

Accordingly, understanding students’ adoption of e-learning is imperative to enable universities to establish a supportive infrastructure and to design effective e-learning programs.

The adoption of e-learning has been proven to be a reliable proxy for the success of any information technology-based initiative in the education sector [10]. This study aimed at determining the predictors for the adoption of e-learning among health professional students during the Coronavirus diseases 2019 lockdown at a private university in Uganda. The study adopted a Technology Acceptance Model (TAM) to understand whether health professional students who were introduced to new technology, accept it considering their perceived ease of use and value for their education [11] in the form of learner and Information Technology (IT) aspects.

## Methods

### Study design

The study employed a cross-sectional study design where quantitative data were collected from participants electronically. Emails were only sent to students that were health professionals. Data on learner aspects (age, gender, time management, e-learning expectations), and IT aspects (confidence in using IT and online communication tools, prior experience with e-learning, flexibility of e-learning and internet cost) were obtained for the purpose of comparing differences in adoption of e-learning.

### Setting and study participants

The study was conducted at Clarke International University (CIU). CIU is one of the private universities located in Kampala city with a population of nearly 1000 students of which approximately 150 are health professionals. E-learning at CIU progressively became popular during the national Coronavirus diseases 2019 lockdown [12]. Before the pandemic, e-learning was only restricted to postgraduate students taking a Master’s in Public Health with a blended approach. Currently, e-learning at CIU has been integrated into all university programs both at undergraduate and postgraduate levels.

Continuing health professional students aged ≥18 years at Clarke International University by the time of the lockdown in Uganda (18th March 2020) were included in the study. Participants included continuing students doing Diploma courses in Clinical Medicine and Pharmacy, Postgraduate Diplomas in Medical Education, Bachelor’s in Medical Laboratory Science, and Bachelor’s in Nursing and Midwifery. An email was sent to the students with a brief description of the study asking for their willingness to participate. E-learning was defined as a virtual learning system supported by the use of computers or smartphones and internet use. Students who were willing to participate were later sent an electronic questionnaire with consent. Students could only proceed to the questions after consenting.

The following categories of health professional students were excluded from the study: 1) students in their final year of study who were only left with research to complete the program, and 2) students who were enrolled on remote learning before the lockdown. Data was collected over a period of 3 weeks.

### Measurements

#### Outcome variable

Our outcome variable (adoption of e-learning) was measured by self-report on a binary scale. Respondents were asked whether they were satisfied with the decision to continue taking the course online, whether e-learning would satisfy their learning needs and if they would gladly take another program fully via the internet in any institution of higher learning given an opportunity. Responses were later categorized as a binary outcome (Yes- adopted e-learning and No- did not adopt e-learning).

#### Independent variables

The independent variables included learner aspects (age, gender, time management, e-learning expectations); and IT aspects (confidence in using IT and online communication tools, prior experience with e-learning, flexibility of e-learning and internet cost).

We collected quantitative data using a smart survey where participants accessed the survey through their emails. This enabled ease of questionnaire distribution and collection of survey data in real-time**.** Prior to data collection, the questionnaire was pre-tested outside the study area (Kampala International University). Unique identifiers like email and names of participants were removed before data analysis to ensure confidentiality of the collected data.

### Data analysis

We analyzed data using IBM Statistical Package for Social Sciences (SPSS) version 20 predictive analytics software. In the descriptive analysis of categorical data, frequencies and percentages were summarized. In the bivariate analysis, we conducted the Chi-square test for differences in e-learning adoption as long as all the cell counts were ≥ 5 and Fisher’s exact test was used for age, high expectations in e-learning and affordable internet since their cell counts were < 5. In the multivariate analysis, all variables with a probability value (*p*-value) less than 5% at bivariate analysis and statistically significant variables from the literature particularly age and sex were included in the regression model. Consequently, we computed crude odds ratio (cOR) and adjusted odds ratio (aOR) at multivariate analysis using binary logistic regression. We reported aORs with the corresponding 95% confidence interval (CI). We did not report probability values (*p*-values) since Confidence Intervals (CIs) are satisfactory for recording the precision of the measure of effect and establishing statistical significance [13]. Furthermore, CIs are more enlightening than probability values [14] and are henceforth preferred in reporting the outcome [15].

## Results

A total of 109 (response rate 91.6%) continuing health professional students at CIU participated in the study. The study included those who had not completed their courses by the time of the first National Coronavirus diseases 2019 lockdown on the 18th of March 2020. Students who had initially applied for the remote learning program before the national lockdown were excluded from the study.

### General characteristics of participants

Of the 109 participants, 65 (59.6%) were female, 69 (63.3%) were in the 20–29 years age bracket, 63 (57.8%) were in their first year of study, 68 (62.4% were single and 57 (52.3%) were not in the frontline of COVID 19 management.

### Differences in the adoption of e-learning with the learner and IT aspects

The differences in the adoption of e-learning with learner aspects and IT aspects are shown in Table 1. Overall, 72 (65.1%) adopted e-learning during the COVID 19- Lockdown. E-learning adoption was more prevalent among female health professional students (67.7%), participants aged 30–39 years (72.7%), in the first year of study (77.8%), in union/married or cohabiting (75.6%), and in the frontline of COVID 19 management (66.7%). We observed statistically significant differences in e-learning adoption concerning year of study, time management, high e-learning expectations, confidence in using IT devices, Prior experience in e-learning, considering e-learning to be flexible and affordable internet.

**Table 1:** Bivariate analysis of differences in e-learning adoption with the learner and IT aspects

	Total	E-learning adoption		*p*-value
	Overall *n* = 109	No (34.9%) *n* = (38)	Yes (65.1%) *n* = (71)	
**Sex**				
Female	65 (59.6)	21 (32.3)	44 (67.7)	0.496
Male	44 (40.4)	17 (38.6)	27 (61.4)	
**Age**				
20–29 years	69 (63.3)	26 (37.7)	43 (62.3)	1.868
30–39 years	33 (30.3)	9 (27.3)	24 (72.7)	
40 and above	7 (6.4)	3 (42.9)	4 (57.1)	
**Year of study**				
One	63 (57.8)	14 (22.2)	49 (77.8)	0.002
Two	46 (42.2)	24 (52.2)	22 (47.8)	
**Marital status**				
In union	41 (37.6)	10 (24.4)	31 (75.6)	0.054
Not in union	68 (62.4)	28 (41.2)	39 (58.8)	
**In COVID 19 management frontline**				
No	52 (47.7)	19 (36.5)	33 (63.5)	0.807
Yes	57 (52.3)	19 (33.3)	38 (66.7)	
**I manage time well**				
No	37 (29.4)	18 (48.6)	19 (51.4)	0.030
Yes	72 (70.6)	20 (27.8)	52 (72.2)	
**High expectations in e-learning**				
No	9 (8.3)	8 (89.0)	1 (11.0)	0.001
Yes	100 (91.7)	30 (30.0)	70 (70.0)	
**I feel confident using IT devices**				
No	32 (29.4)	19 (59.4)	12 (40.6)	< 0.001
Yes	77 (70.6)	19 (24.4)	59 (75.6)	
**Prior experience in e-learning**				
No	30 (27.5)	18 (60.0)	12 (40.0)	0.001
Yes	79 (72.5)	20 (25.3)	59 (74.7)	
**I consider e-learning to be flexible**				
No	31 (28.4)	17 (54.8)	14 (45.2)	0.006
Yes	78 (71.6)	21 (26.9)	57 (73.1)	
**Affordable internet**				
No	73 (67.0)	32 (43.8)	41 (56.2)	0.005
Yes	36 (33.0)	6 (16.7)	30 (83.3)	

### Factors associated with the adoption of e-learning at the unadjusted and adjusted analysis

Table 2 shows low odds of adoption of e-learning among participants in their first year of study (aOR, 0.34: 95%CI, 0.14–0.79), low e-learning expectations (aOR, 0.01: 95%CI, 0.01–0.34), no confidence in using IT devices (aOR, 0.16: 95%CI, 0.00–0.77), prior use of e-learning tools (aOR, 0.11: 95%CI, 0.02–0.68), not considering e-learning flexible (aOR, 0.25:95%CI, 0.08–0.86) high cost of internet (aOR, 0.13: 95%CI, 0.02–0.84).

**Table 2:** Factors associated with the adoption of e-learning at unadjusted and adjusted analysis

Variable	Binary Logistic Regression			
	Unadjusted analysis		Adjusted analysis	
	OR	95%CI	aOR	95%CI
**Sex**				
Male	0.71	0.32–1.59	0.998	0.27–3.75
Female	1		1	
**Year of study**				
One	0.37	0.17–0.84	0.34	0.14–0.79*
Two	1		1	
**I manage time well**				
No	0.41	0.18–0.93	0.62	0.16–2.47
Yes	1		1	
**I have high expectations in e- learning**				
No	0.05	0.01–0.45	0.01	0.01–0.34*
Yes	1		1	
**I feel confident using IT devices**				
No	0.20	0.08–0.50	0.16	0.00–0.77*
Yes	1			
**Prior experience in e-learning**				
No	0.23	0.09–0.55	0.11	0.02–0.68*
Yes	1		1	
**Consider e-learning to be flexible**				
No	0.30	0.13–0.72	0.25	0.08–0.86*
Yes	1		1	
**Affordable internet**				
No	0.26	0.10–0.69	0.13	0.02–0.84*
Yes	1		1	

Significance codes at 5% level: *p* < 0.001***, *p* < 0.01**, *p* < 0.05*

## Discussion

The aim of this study was to determine predictors for the adoption of e-learning among health professional students at a private university during the Coronavirus diseases 2019 lockdown. The data show that 65.1% of continuing health professional students adopted e-learning. This finding is consistent with a recent study in Ghana [16] where university students had a positive attitude toward e-learning during the lockdown. However, the rate of e-learning adoption in the current study was twice and five times higher than in a comparative study done in Nigeria (32.5%) and Uganda (14.4%) respectively [17]. The discrepancy could be attributed to the fact that the previous studies were conducted in Public Universities with a high volume of students compared to the current study setting which is a private university with relatively low numbers of students that are health professionals. Our results suggest that private universities’ transition from face-to-face teaching to e-learning during the lockdown was relatively smooth albeit with some challenges. In comparison, documented experiences of public universities’ transition to e-learning reflect equal if not more challenges in the adoption of e-learning [18]. Accordingly, both private and public universities in Uganda must develop strategies to ensure that all students continue to learn during the lockdown and beyond any unprecedented time.

Our study shows that health professional students in their first year of study are less likely to adopt e-learning compared to those in the second year. Though no study has directly linked e-learning adoption to a year of study, a previous study [19] revealed that IT adoption in universities is incremental. This could imply that first-year health professional students have not adequately utilized IT in education. Therefore, adopting e-learning would be slower process compared to advanced students who had more previous exposure to e-learning.

This could be due to the low anticipation of what e-learning can contribute towards continuity of learning especially during such unprecedented times of coronavirus diseases 2019. This finding is consistent with King and Boyatt’s [20] study exploring factors influencing the adoption of e-learning in higher education where adoption of e-learning was found to be dependent on student expectations. Additionally, Kirkwood [21] in his review of evidence related to e-learning and higher education practices argues that e-learning does not, in itself, result in improved educational outcomes; in some instances, students were concerned about the teaching methodology and assessment which may explain their hesitance to adopt e-learning.

Our finding that students with low confidence in using IT devices are less likely to adopt e-learning is not surprising. This is because e-learning is largely premised on the presence of IT and related infrastructure. In a recent integrative review, O’Doherty et al., [22] reported that some of the challenges of implementing e-learning in medical education were issues related to IT (poor technical skills, inadequate infrastructure, absence of institutional support, negative attitudes…). This implies that overall IT improvement is an integral part of introducing e-learning in any academic institution.

Our data also show that students with no prior e-learning experience are less likely to adopt e-learning. Previous studies [23, 24] have documented that having prior e-learning experience exposes students to some level of online skills and online system competency. In their study assessing dental students’ readiness to adopt e-learning, Linjawi and Alfadda [23] reported that students’ readiness to adopt was related to acceptable levels of online system competency. Relatedly, the acceptability to adopt technology has been cited as largely depending on prior use or recommendations from previous users [24]. Institutional approaches such as blended learning [25, 26] that expose learners to e-learning systems to enable continuous adoption and utilization of e-learning in the future are necessary.

We found that students who did not consider e-learning a flexible way to study were less likely to adopt e-learning. Previous studies [27] have demonstrated flexibility as an additional positive feature of e-learning; emphasizing that a student can plan their time for completion of available courses virtually. However, robust orientation, sensitization, and training to enhance digital literacy are necessary to improve attitudes and the adoption of e-learning systems [7].

In this study, participants who found the cost of the internet unaffordable were less likely to adopt e-learning during the lockdown. This finding is consistent with previous studies in South Sudan where relatively expensive out-of-pocket spending on internet bundles affected the continuity of learning in medical schools [28]. Previous studies have also linked e-learning success to the reliability of the internet [7, 29] where unreliable internet affected student adoption of e-learning. This clearly implies that access to the internet is a necessary factor for students’ successful adoption of e-learning. Although students in high-income countries had the ability to transition from traditional face-to-face education to e-learning during the Coronavirus diseases 2019 pandemic, students in low- and middle-income countries like Uganda, where access to the internet is not free, might struggle to fully adopt online education [30]. Therefore, it is imperative for universities to advocate for internet policy reforms that address the cost of acquisition of ICT equipment, zero-rating educational content on the internet, and incentivizing telecom companies for special contracts that enhance internet access for learners to accelerate the adoption of e-learning during the Coronavirus diseases 2019 pandemic and beyond [29–31].

This study also confirms the applicability of TAM where students who have been introduced to new technology can accept and use it based on perceived easiness to use and flexibility.

### Study limitations

Limitations of the study include the lack of data on the physical parameters. Additionally, we used a cross-sectional design so we can only show association and not causation. Another limitation is the lack of qualitative data to explain some of the reasons for the adoption of e-learning. More so, the group of students who were surveyed is relatively small and the study presents data from only one institution. Many additional factors that were not tested in this study may influence the adoption of e-learning by students. These limitations should be considered in the interpretation of the results.

## Conclusions

Our study shows that predictors of e-learning adoption are year of study, high e-learning expectations, confidence in using IT devices, prior experience of e-learning, considering e-learning to be a flexible way to study and affordable internet. Our findings suggest a need to prepare students intending to engage in online learning on costs related to internet activity and internet bundles and offer ICT training to all potential e-learners for easy adoption.

## Supplementary Information

**Additional file 1.**

## Data Availability

The dataset for the current study is available and can be provided by the corresponding author upon reasonable request.

## Abbreviations

aORs: Adjusted odds ratios
CI: Confidence Interval
CIU: Clarke International University
cORs: Crude odds ratios
E-learning: Electronic learning
ICTs: Information Communication Technologies
IT: Information Technology
NCHE: National Council for Higher Education
TAM: Technology Acceptance Model
SPSS: Statistical Package for the Social Sciences
