# Supplementary material for: Predictors for adoption of e-learning among health professional students during the COVID-19 lockdown in a private university in Uganda
Source: BMC Med Educ. 2022 Sep 10;22:671. doi: 10.1186/s12909-022-03735-7 (PMC9463679; doi:10.1186/s12909-022-03735-7)
Supplement: Supplementary file 1 — Additional file 1. [file 12909_2022_3735_MOESM1_ESM.pdf]

Appendix ii: Questionnaire

Title: Predictors for adoption of e-learning among health professional students during the COVID-19 lockdown in a Private University in Uganda.

Section A: Characteristics of Respondents

In this section, you are required to give your response by ticking the appropriate response. Only question 2 requires that you provide the answer on the space provided.

S/N	Question	Response	Codes
1	What is your sex?	Male<br>Female	[1]<br>[2]
2	How old are you?	______________	
3	What is your year of study?	Year 1<br>Year 2	[1]<br>[2]
4	What is your marital status?	Single<br>Married/cohabiting<br>Separated/divorced<br>Widowered	[1]<br>[2]<br>[3]<br>[4]
5	In the frontline of COVID 19 management	Yes<br>No	[1]<br>[2]

Section B: Learner perspectives and adoption to E-learning
In this section, you are required to give your response by ticking only one response;

**S/N**	**Question**	Response
	**Learner component of the E-Learning Environment**	
6	Are you able to carry out my own study plan	Yes [1]<br>No [2]
7	Do you seek assistance when you face learning problems?	Yes [1]<br>No [2]
8	Are you able to manage time well?	Yes [1]<br>No [2]
9	Did you set up your own learning goals?	Yes [1]<br>No [2]
10	Do you high expectations for e-learning?	Yes [1]<br>No [2]
11	Can you direct your own learning progress?	Yes [1]<br>No [2]
12	Are you distracted by other online activities when learning on line?	Yes [1]<br>No [2]
13	Do you repeat the instruction materials on the basis of my needs?	Yes [1]<br>No [2]
14	Do you feel confident in performing the basic functions of Microsoft office programs?	Yes [1]<br>No [2]
15	Do you feel confident in your knowledge and skills of how to manage software for online learning?	Yes [1]<br>No [2]
16	Do you feel confident using the internet to find or gain information on line?	Yes [1]<br>No [2]
17	Do you feel confident in using online tools (emails, discussion forums) to effectively communicate with others?	Yes [1]<br>No [2]

18	Do you feel confident in expressing myself (emotion, humor) through text?	Yes [1]<br>No [2]
19	Do You feel confident in posting questions in online discussion?	Yes [1]<br>No [2]
	**Technology Perspectives**	
	**For QN 20-23**<br>**Are the information technologies used in e-learning…….**	
20	are very easy to use	Yes [1]<br>No [2]
21	have many useful functions	Yes [1]<br>No [2]
22	have good flexibility	Yes [1]<br>No [2]
23	are easy to obtain	Yes [1]<br>No [2]
24	Do you have any issues with the internet speed?	Yes [1]<br>No [2]
25	Is it easy to go online?	Yes [1]<br>No [2]
26	Is the fee to connect to internet affordable?	Yes [1]<br>No [2]
27	Are you able to connect to internet whenever you wish?	Yes [1]<br>No [2]
28	Are you familiar with all the E-tools on Moodle platform?	Yes [1]<br>No [2]

THANK YOU FOR PARTICIPATING IN THE STUDY
